# RNA-Based CTC Analysis Provides Prognostic Information in Metastatic Breast Cancer

**DOI:** 10.3390/diagnostics11030513

**Published:** 2021-03-14

**Authors:** Areti Strati, Michail Nikolaou, Vassilis Georgoulias, Evi S. Lianidou

**Affiliations:** 1 Analysis of Circulating Tumor Cells Lab, Department of Chemistry, University of Athens, 15771 Athens, Greece; astrati@chem.uoa.gr; 2 Medical Oncology Unit, “Elena Venizelou” Hospital, 11521 Athens, Greece; nikolaoumike@hotmail.com; 3 Metropolitan General Hospital, 15562 Athens, Greece; georgulv@otenet.gr

**Keywords:** liquid biopsy, circulating tumor cells, gene expression, metastatic breast cancer, multiplex RT-qPCR, prognostic marker

## Abstract

In metastatic breast cancer (MBC) the molecular characterization of circulating tumor cells (CTCs) provides a unique tool to understand metastasis-biology and therapy-resistance. We evaluated the prognostic significance of gene expression in EpCAM^(+)^ CTCs in 46 MBC patients based on a long follow-up. We selected a panel consisting of stem cell markers (*CD24*, *CD44*, *ALDH1*), the mesenchymal marker *TWIST1*, receptors (*ESR1*, *PGR*, *HER2*, *EGFR*) and the epithelial marker *CK-19*. Singleplex RT-qPCR was used for *TWIST1* and *CK-19* and multiplex RT-qPCR for stem cell markers and receptors. A group of 19 healthy donors (HD) was used as control. Univariate (*p* = 0.001) and multivariate analysis (*p* = 0.002) revealed the prognostic value of combined gene expression of *CK-19*(+), *CD44^high^/CD24^low^*, *ALDH1^high^/CD24^low^* and *HER2* over-expression for overall survival (OS). The Kaplan–Meier estimates of OS were significantly different in patients positive for *CK-19* (*p* = 0.028), *CD44^high^/CD24^low^* (*p* = 0.002), *ALDH1^high^/CD24^low^* (*p* = 0.007) and *HER2*-positive (*p* = 0.022). Our results indicate that combined gene expression analysis in EpCAM^(+)^ CTCs provides prognostic information in MBC.

## 1. Introduction

Circulating tumor cells (CTCs) are rare, heterogeneous and difficult to analyze, but constitute highly important players in liquid biopsy [1,2,3] since rational treatment decisions and monitoring therapeutic response could be based on the identification of cancer specific biomarkers in these cells [4]. CTC enumeration in the CellSearch provides prognostic information in metastatic breast, prostate and colorectal cancer and is the only FDA-cleared assay up to now [5,6].

In addition to enumeration, the molecular characterization of CTC both at a bulk and single cell level [7,8] reveals tumor heterogeneity and can give valuable information on cancer evolution in real time [7,9,10]. CTC analysis at the RNA level could provide important prognostic information and represents an innovative and promising approach in the clinical management of cancer patients [11]. There is now evidence showing that RNA analysis in CTCs of patients with metastatic castration resistant prostate cancer (mCRPC) at several time-points can demonstrate a continuous change in the expression of *AR-V7* splice variant and this has already been connected with response to chemotherapy and androgen deprivation therapy [12,13]. The prognostic significance of *CK-19* positive CTCs has already been shown in early and metastatic breast cancer (MBC) [14]. There is evidence that the expression of a variety of markers in CTCs can change during systemic treatment of MBC patients [15,16], and specific gene signatures of CTCs in breast cancer are correlated with the risk for brain metastasis [4]. The presence of mutations in specific genes like *PIK3CA* or *ESR1*, or epigenetic alterations in CTCs can define specific subgroups of patients suitable for targeted therapy [17,18]. 

In primary tumors, it has been shown that subpopulations of tumor cells that display the CD44^high^/CD24^low^ profile and express aldehyde dehydrogenase 1 (ALDH1+) are of high tumorigenic potential [19]. The putative stem cell phenotype (CD44+/CD24− and/or ALDH1+/CD24−) has been detected in CTCs [20], and the tumor microenvironment regulates the plasticity of cancer stem cells through transition between epithelial to mesenchymal-like (EMT) and mesenchymal to epithelial-like (MET) states [21]. Based on molecular analysis in EpCAM^(+)^ CTCs at the RNA level, we have previously shown that in early stage breast cancer, there is a significantly higher risk of relapse and death in patients expressing both stem cell and mesenchymal characteristics [22].

Various studies have shown that the expression of *ER*, *PR* and *HER2* in CTCs may change during the course of the disease [23]. Differences reported in *HER2* amplification between CTCs and the primary tumor [24] suggest that *HER2* amplification in CTCs could add important information for HER2-targeted therapy [25]. In addition to HER2 amplification, the detection of *HER2* overexpression in CTCs at the RNA level, is able to predict the HER2 status on metastases [23]. It has also been shown in patients with HER2-positive breast cancer that the expression of truncated HER2 on CTCs is associated with poor survival and could be related to resistance to trastuzumab [26]. On the other hand, the effectiveness of endocrine therapy in patients with hormone receptor (HR)-positive breast cancer is limited by high rates of de novo resistance and acquired resistance during treatment [27]. Therefore, real-time monitoring of ER/PR status in CTCs may help to understand the bases of resistance to endocrine treatment [28].

In the present study we report on the prognostic significance of combined gene expression analysis in EpCAM^(+)^ CTCs in MBC patients using a panel consisting of the epithelial marker *CK-19*, a panel of receptors (*ESR1*, *PR*, *HER2*, *EGFR*), the mesenchymal marker *TWIST1* and stem cell markers (*CD24*, *CD44*, *ALDH1*).

## 2. Materials and Methods 

### 2.1. Cell Lines

The human mammary carcinoma cell line SKBR-3 was used as a positive control for *CD24*, *CD44*, *ALDH1*, and *HPRT* expression [22]. SKBR-3 and T47D cancer cell lines were used as a positive control for the development of the quadraplex RT-qPCR assay for *ESR1*, *PR*, *HER2*, and *EGFR* expression. MCF-7 cells were used as a positive control for *CK-19* expression, while for *TWIST1* expression MDA-MB-231 cells were used [22]. Cells were counted in a hemocytometer and their viability was assessed by trypan blue dye exclusion. cDNAs of all these cancer cell lines were kept in aliquots at −20 °C and used as positive controls in parallel to the analysis of patient’s samples. 

### 2.2. Patients

Forty-six patients with already diagnosed MBC were enrolled in the Medical Oncology Unit of “Elena Venizelou” Hospital from July 2009 until February 2011, and their clinical characteristics at the time of diagnosis are shown in Table 1. Peripheral blood was collected from all patients when metastasis was verified and before 1st line of treatment. The median follow-up period since primary tumor diagnosis was around 8 years (mean + SD: 7.7 + 4, median: 8 years). All patients signed an informed consent to participate in the study which was approved by the Ethics and Scientific Committees of our Institution. A group of 19 healthy female blood donors was used as a control group.

### 2.3. Isolation of EpCAM^(+)^ CTCs, RNA Extraction and cDNA Synthesis

Peripheral blood (10 mL in EDTA) from patients and healthy donors (HD) was collected and processed within 3h in exactly the same manner. All steps including, the isolation of EpCAM^(+)^ cells, RNA extraction and cDNA synthesis were performed as previously described [22].

### 2.4. RT-qPCR

Singleplex RT-qPCR assays were used for the epithelial marker *CK-19* [29] and the mesenchymal marker *TWIST1* [22] as previously described. A quadraplex RT-qPCR was used for the stem cell markers (*CD24*, *CD44*, *ALDH1*, and *HPRT*) as recently described [22]. For *ESR1*, *PR*, *HER2*, and *EGFR* we developed and validated a novel quadraplex RT-qPCR assay. Each probe set included a 3’-fluorescein (F) donor probe and a 5’-LightCycler (LC) acceptor probe that was different for each gene set: *ESR1*: at 610 nm, *PR*: at 640 nm, *HER2*: at 670 nm, *EGFR*: at 705 nm. A color compensation test was performed by using pure dye spectra so that spectral overlap between dyes was corrected [16]. Component concentrations and the cycling conditions of the quadraplex RT-qPCR assay were optimized in detail. The amplification reaction mixture (10 μL) for the *ESR1*, *PR*, *HER2*, *EGFR* multiplex assay contained 1 μL of the PCR Synthesis Buffer (5Χ), 1.6 μL of MgCl_2_ (25 mM), 0.2 μLdNTPs (10 mM), 0.2 μL BSA (10 μg/μL), 0.1 μL Hot Start DNA polymerase (HotStart, 5 U/μL, Promega, USA), 0.6 μL of a mixture containing all eight primers (10 μΜ), 0.5 μL of a mixture containing all eight dual hybridization probes (3 μM) and H_2_O (added to the final volume). Cycling conditions were: 95 °C/2 min; 45 cycles of 95 °C/20 s, annealing at 58 °C/20 s and extension at 72 °C/20 s. For the development and analytical evaluation of the assay, we generated individual PCR amplicons corresponding to the four gene-targets studied that would serve as quantification calibrators, as we have previously described [16,22]. All RT-qPCR reactions were performed in the LightCycler 2.0 (Roche, Germany) and for every RT-qPCR assay we set a cut-off value following strict criteria. In cases of *CK-19* and *EGFR* we did not estimate any cut-off value since these genes are not expressed in PBMCs of HD. For all other genes studied we estimated the cut off value for genes that were also expressed in HD samples (due to the background noise of PBMC co-isolated with CTCs) based on the mean Cq value and the standard deviation for each gene in HD samples. Thus the average + 2 SD was used as the upper limit of normal background expression (with 95% confidence) [22,30]. 

### 2.5. Statistical Analysis

Statistical analysis was performed using SPSS (SPSS Statistics 25.0). Mann–Whitney U test, was used in order to evaluate the differences in gene expression between cancer patients and healthy individuals. Associations between gene expression markers and other clinicopathological variables of the MBC patients were analyzed using the Fisher’s exact test. The overall survival (OS) rate was calculated by the Kaplan-Meier method and was evaluated by the log-rank test. OS was defined as the time from sample collection and thus registration to the study to death from any cause or censored at the time of last contact. Univariate COX regression analysis was conducted to estimate the prognostic utility of gene expression markers for the OS of MBC patients. Multivariate Cox proportional hazards models were used to evaluate the relationship between gene expression status and event-time distributions, with tumor size, grade, number of involved lymph nodes, ER, PR, HER2 and age. All *p*-values are two-sided. A level of *p* < 0.05 is considered statistically significant.

## 3. Results

### 3.1. Combined Gene Expression Analysis in EpCAM^(+)^ CTCs

**Figure 1:**
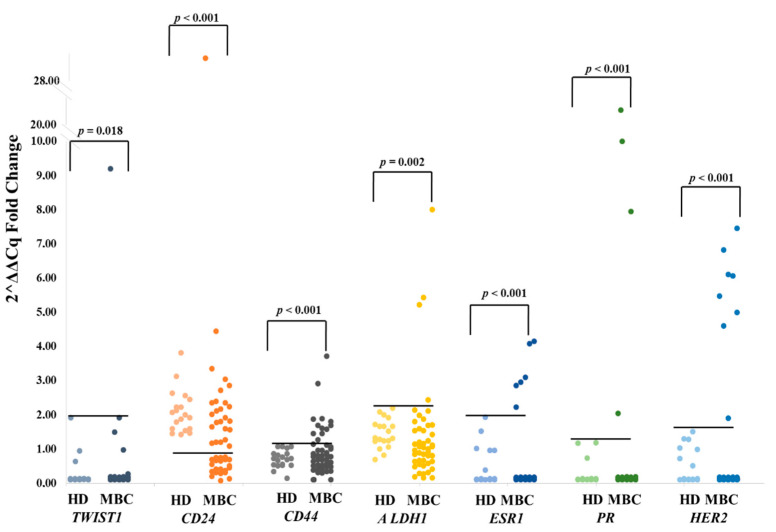
*TWIST1*, *CD24*, *CD44*, *ALDH1*, *ESR1*, *PR* and *HER2* expression in EpCAM^(+)^ CTC fraction from MBC patients (*n* = 46) and HD (*n* = 19), (relative fold change values, 2^–ΔΔCq^ in respect to *HPRT* expression).

We quantified *CK-19*, *ESR1*, *PR*, *HER2*, and *EGFR* transcripts and evaluated the stem cell profiles *CD44^high^/CD24^low^* and *ALDH1^high^/CD24^low^* and the mesenchymal marker *TWIST1* in EpCAM^(+)^ CTCs in all patient samples (Figure 1). Prior to proceeding to combined gene expression analysis, the quality of all cDNAs was checked through RT-qPCR for *HPRT* (reference gene). We define an EpCAM^(+)^ CTC fraction as positive or negative for the expression of a specific gene, based on the RT-qPCR results, and the detection of this gene transcript in the HD control group (analyzed in exactly the same way). *CK-19* and *EGFR* transcripts were quantified based on the absolute quantification approach since no transcripts of these genes were detected in the EpCAM^(+)^ fraction of HD. Based on that, all samples showing amplification curves in RT-qPCR for *CK-19* and *EGFR* were positive. *ESR1*, *PR*, *HER2*, *CD24*, *CD44*, *ALDH1*, *TWIST1* transcripts were quantified based on the relative quantification approach, since these transcripts were also detected in the EpCAM^(+)^ fraction of HD samples (analyzed in exactly the same way). More specifically:

#### 3.1.1. *TWIST1*

Only in 1/46 (2.2%) sample *TWIST1* was overexpressed (*TWIST1^high^*, median fold change: 9.19), while in 45/46 (97.8%) samples *TWIST1* expression was low and similar to HD (*TWIST1^low/-^*, median fold change: 0.0, range: 0–1.91) (Mann–Whitney test, *Ζ* = −2.357, *p* = 0.018). In HD the median relative fold change of *TWIST1* expression in respect to *HPRT* expression in the EpCAM^(+)^ fraction was 0.0 (range: 0–2.0).

#### 3.1.2. *CD24*

21/46 (45.7%) EpCAM^(+)^ samples were found to express low levels of *CD24* (*CD24^low^*). Median fold change of *CD24* expression in respect to *HPRT* expression in the EpCAM^(+)^ fraction was 2.00 (range: 1.42–3.81) in HD and 0.45 (range: 0.12–0.75) in these 21 *CD24^low^* patients’ samples, while it was 2.09 (range: 1.08–29.65) in the remaining 25 patients’ samples (Mann–Whitney test, *Ζ* = −5.405, *p* < 0.001). 

#### 3.1.3. *CD44*

12/46 (26.1%) EpCAM^(+)^ samples were positive for *CD44* overexpression (*CD44^high^*). Median fold change of *CD44* expression in respect to *HPRT* expression in the EpCAM^(+)^ fraction was 0.71 (range: 0.14–1.06) in HD and 1.69 (range: 1.27–3.71) in *CD44^high^* and 0.62 (range: 0.0–1.14) in the remaining 34 patients’ samples (Mann–Whitney test, *Ζ* = −4.627, *p* < 0.001). 

#### 3.1.4. *ALDH1*

4/46 (8.7%) EpCAM^(+)^ samples were positive for *ALDH1* overexpression (*ALDH1^high^*). Median fold change of *ALDH1* expression in respect to *HPRT* expression in the EpCAM^(+)^ fraction was 1.32 (range: 0.69–2.19) in HD and 5.32 (range: 2.43–8.00) in *ALDH1^high^* and 0.93 (range: 0.18–2.14) in the remaining 42 patients samples (Mann–Whitney test, *Ζ* = −3.086, *p* = 0.002). 

#### 3.1.5. *ESR1*

6/46 (13.0%) EpCAM^(+)^ samples were positive for *ESR1* overexpression. Median fold change of *ESR1* expression in respect to *HPRT* expression in the EpCAM^(+)^ fraction was 0.0 (range: 0.0–1.92) in HD and 3.00 (range: 2.22–4.14) in *ESR1^high^* and 0.0 (range: 0.0–0.0) in the remaining 40 patients’ samples (Mann–Whitney test, *Ζ* = −3.911, *p* < 0.001).

#### 3.1.6. *PR*

4/46(8.7%) EpCAM^(+)^ samples were positive for *PR* overexpression. Median fold change of *PR* expression in respect to HPRT expression in the EpCAM^(+)^ fraction was 0.0 (range: 0.0–1.18) in HD and 8.97 (range: 2.04–21.6) in *PR^high^* and 0.0 (range: 0.0–1.42) in the remaining 42 patients’ samples (Mann–Whitney test, *Ζ* = −3.782, *p* < 0.001).

#### 3.1.7. *HER2*

8/46 (17.4%) EpCAM^(+)^ samples were positive for *HER2* overexpression (*HER2^high^*). Median fold change of *HER2* expression in respect to *HPRT* expression in the EpCAM^(+)^ fraction was 0.28 (range: 0.0–1.49) in HD and 5.76 (range: 1.90–7.45) in *HER2^high^* and 0.0 (range: 0.0–0.65) in the remaining 38 patients’ samples (Mann–Whitney test, Ζ = −4.112, *p* < 0.001).

#### 3.1.8. *EGFR*

*EGFR* transcripts in all EpCAM^(+)^ samples were quantified by RT-qPCR by absolute quantification. All samples both in the group of HD and MBC patients were found negative for *EGFR* expression in the EpCAM^(+)^ fraction. 

#### 3.1.9. *CK-19*

*CK-19* transcripts in all samples were quantified by RT-qPCR by absolute quantification. No transcripts were detected in the EpCAM^(+)^ fraction of HD (0/19, 0%). Conversely, 10/46 (21.7%) EpCAM^(+)^ samples were found positive for *CK-19* expression.

**Figure 2:**
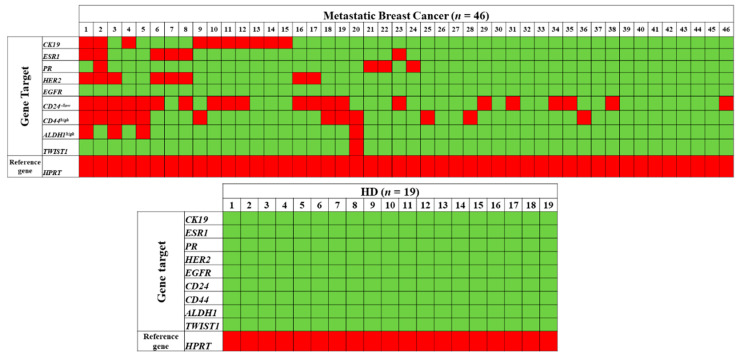
*CK-19*, *ESR1*, *PR*, *HER2*, *EGFR*, *CD24*, *CD44*, *ALDH1* and *TWIST1* expression in the EpCAM^(+)^ CTC fraction from MBC patients (*n* = 46) and HD (*n* = 19). Red color represents detection or overexpression, while green color indicates no detection or underexpression.

Our results are based on bulk CTC analysis, so by using immune-magnetic beads targeting the epithelial antigen EpCAM we are actually not only enriching our samples with CTCs but we also co-isolate a low fraction of non-specifically bound PBMCs. The presence of these non-specific cells is verified by expression of *HPRT* in all our EpCAM^(+)^ fractions (Figure 2). *HPRT* expression is used as an internal control for sample quality to avoid false negative results, but also as a reference gene for relative quantification, since it is expressed in all cells, both EpCAM^(+)^ and PBMC. Following this procedure and the identical analysis of HD peripheral blood samples we define a sample as CTC-positive based on the expression of these specific genes that differentiate CTCs from PBMC. When we evaluated a sample as CTC-positive, based on the expression of one of the nine genes studied individually in the EpCAM^(+)^ fraction, a positivity rate ranging between 2.2% and 21.7% was obtained, e.g., CTC positivity rate based on *CK-19* was 21.7% (10/46). However, a significant increase in the positivity rate was observed when we evaluated a sample as CTC-positive, based on the combined expression of at least one of these genes in the EpCAM^(+)^ fraction; in this case the cumulative positivity rate was 52.2% (24/46) (Fischer-Exact test, *p* = 0.010) (Table S1). 7/46 (15.2%) MBC patients’ EpCAM^(+)^ samples were positive for *CD44^high^/CD24^low^* (stem cell profile) and 3/46 (6.5%) were positive for *CD24^low^/ALDH1^high^* (stem cell profile). A representative heat map of gene expression in EpCAM^(+)^ fractions of all samples and HD is shown in Figure 2.

### 3.2. Comparison between HER2 and ER/PR Status of EpCAM^(+)^ CTCs and the Primary Tumor

For 43 out of these 46 patients the ER, PR, and HER2 status of the primary tumor at the time of initial diagnosis was known. 34/43 (79.1%) primary tumors were ER^+^, 30/43 (69.8%) PR^+^ and 9/43 (20.9%) HER2^+^. The concordance rate between *ESR1***,** *PR*, *HER2* expression on EpCAM^(+)^ CTC fractions and the primary tumor was 27.9%, 34.9% and 65.1% respectively (Table S2). The high discrepancy observed could be explained by the fact that HR status and HER2 positivity was compared between the primary tumor at the time of diagnosis and CTCs at the time of metastasis verification. There were only four cases positive for ESR1 both in the primary tumor and EpCAM^(+)^ CTCs, three cases positive for PR both in the primary tumor and EpCAM^(+)^ CTCs, while all samples that were positive for *HER2* overexpression in the EpCAM^(+)^ CTC fractions were negative for HER2 amplification in the primary tumor.

### 3.3. Survival Analysis

**Figure 3:**
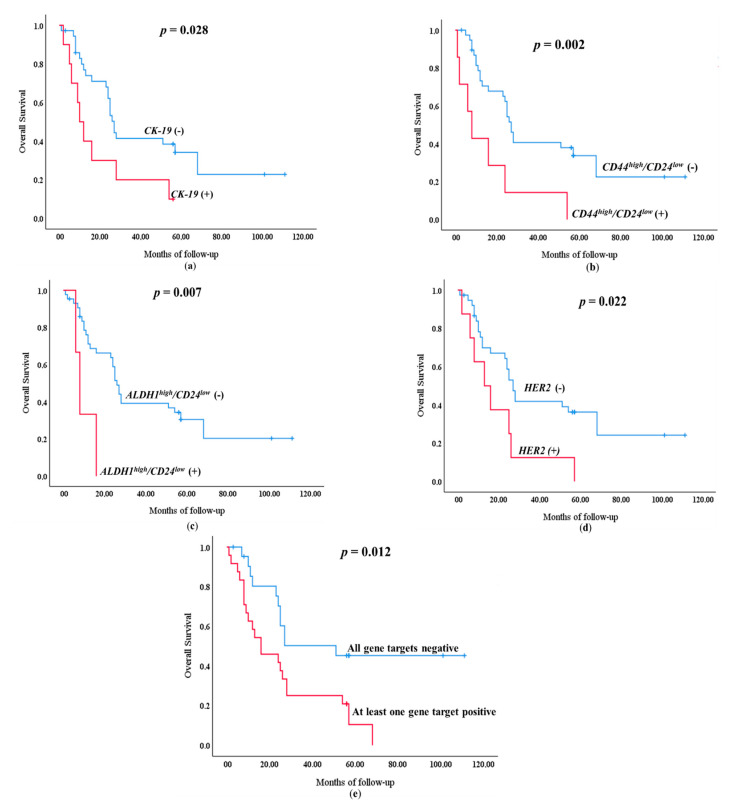
Kaplan–Meier estimates for OS for: (**a**) *CK-19*; (**b**) *CD44^high^/CD24^low^*; (**c**) *ALDH1^high^/CD24^low^*; (**d**) *HER2* overexpression; (**e**) at least one marker positive in the EpCAM^(+)^ CTC fraction.

During the follow up period 32/46 (69.6%) patients died. OS was significantly lower according to Kaplan–Meier estimates in patients positive for *CK-19* (19.8 mo vs. 47.3 mo, *p* = 0.028) (Figure 3A), *CD44^high^/CD24^low^* (15.9 mo vs. 46.7 mo; *p* = 0.002) (Figure 3B), *ALDH1^high^/CD24^low^*(10.0 mo vs. 44.2 mo; *p* = 0.007) (Figure 3C) and *HER2* (19.1 mo vs. 47.5 mo; *p* = 0.022) (Figure 3D), compared to patients who were negative for these gene transcripts in EpCAM^(+)^ CTCs. *TWIST1* (28 mo vs. 42.2 mo; *p* = 0.973), *ESR1* (21.33 mo vs. 44.1 mo; *p* = 0.246) or *PR* (41.1 mo vs. 36.7 mo; *p* = 0.359) positivity in EpCAM^(+)^ CTCs failed to show any statistically significant difference. Moreover, Kaplan Meier analysis revealed that patients positive at least to one marker had lower OS (25.5 mo vs. 62.2 mo; =0.012) when compared to patients that were negative to the expression of all genes tested (Figure 3E).

**Figure 4:**
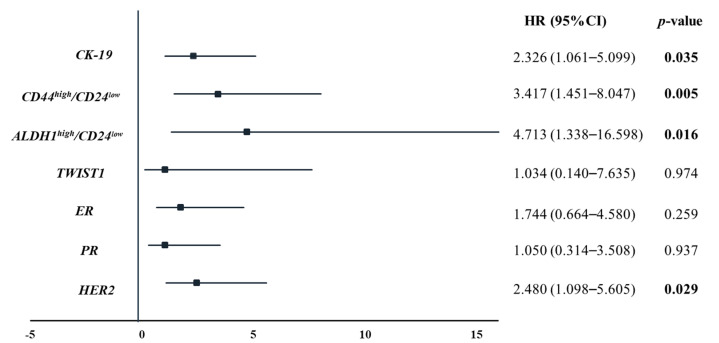
Forest plots of univariate Cox models for OS for MBC patients. Bold values denote statistical significance at the *p* < 0.05 level.

Univariate analysis showed a significantly higher risk of death in the group of patients positive for *CK-19* (HR: 2.326, *p* = 0.035), *CD44^high^/CD24^low^* (HR: 3.417, *p* = 0.005), *ALDH1^high^/CD24^low^* (HR: 4.713, *p* = 0.016) and *HER2*-positive CTCs (HR: 2.480, *p* = 0.029) (Figure 4).

Univariate analysis confirmed the prognostic value of combined gene expression of CK-19, and/or *CD44^high^*/*CD24^low^*, and/or *ALDH1^high^*/*CD24^low^* and/or HER2 in the EpCAM^(+)^ CTC fraction for OS (*p* = 0.001) together with tumor grade (*p* = 0.030) and ER (*p* = 0.004) (Table 2). Multivariate analysis, based on the expression of at least one of the following four profiles: CK-19, and/or *CD44^high^*/*CD24^low^*, and/or *ALDH1^high^*/*CD24^low^* and and/or HER2 independently from patients’ age, tumor T stage, grade, nodal status, and the receptor status (ER, PR, HER2) of the primary tumor confirmed also the prognostic value of gene expression in EpCAM^(+)^ CTCs (*p* = 0.002) (Table 2).

The majority of MBC patients had a bone metastasis (35/46, 76.1%) followed by lung, liver and brain metastasis (10/46, 21.7%) (Table 1). The association of gene expression to bone metastases and other sites of metastasis, is shown in Supplementary Table S3. During the follow-up period 23/35 (35.7%) of MBC patients with bone metastasis died. OS was significantly lower according to Kaplan–Meier estimates in patients with bone metastasis positive for *CK-19* (21.3 mo vs. 49.7 mo, *p* = 0.046) (Figure S1A), *CD44^high^/CD24^low^* (17.4 mo vs. 49.4 mo; *p* = 0.005) (Figure S1B), *ALDH1*^high^/CD24^low^ (6.0 mo vs. 45.9 mo; *p* = 0.002) (Figure S1C) and *HER2* (20.7 mo vs. 50.9 mo; *p* = 0.030) (Figure S1D), compared to patients who were negative for these gene transcripts in EpCAM^(+)^ CTCs. 

The majority of MBC patients received only chemotherapy (37/46, 80.4%) (Table 1). During the follow-up period 27/37 (73.0%) of MBC patients that received only chemotherapy died. OS was significantly lower according to Kaplan–Meier estimates in patients positive for *CK-19* (19.8 mo vs. 37.2 mo, *p* = 0.039) (Figure S2A), *CD44^high^/CD24^low^* (15.9 mo vs. 36.7 mo; *p* = 0.004) (Figure S2B), *ALDH1*^high^/CD24^low^ (10 mo vs. 34.7 mo; *p* = 0.014) (Figure S2C) and *HER2* (19.1 mo vs. 36.9 mo; *p* = 0.041) (Figure S2D), compared to patients who were negative for these gene transcripts in EpCAM^(+)^ CTCs. 

## 4. Discussion

In the present study we report on the prognostic significance of combined gene expression analysis in EpCAM^(+)^ CTCs in MBC patients using a panel consisting of nine genes. It is now evident that molecular characterization of CTC at the RNA level can provide important information on the administration [31] or change of a particular treatment in prostate cancer [32]. Our gene expression analysis was based on a panel of stem cell markers (*CD24*, *CD44*, *ALDH1*), the mesenchymal marker *TWIST1*, a panel of receptors (*ESR1*, *PR*, *HER2*, *EGFR*) and the epithelial marker *CK-19* and was performed according to the established guidelines [33]. Our results clearly indicate that when using a combined RNA analysis based on the expression of these nine genes the positivity rate for CTC presence in the EpCAM^(+)^ fraction was significantly increased in comparison to that derived when CTC detection was based on one or a few genes. 

Kaplan–Μeier analysis showed that the group of patients that were found positive for at least one marker had worse prognosis. Similar to our results, Bredemeier et.al have also shown that the expression of a gene combination in EpCAM^(+)^ CTCs has a negative prognostic effect in MBC patients 8–12 weeks after chemo-, hormone or antibody therapy [34], and Reijm et al. identified an 8-gene CTC predictor which discriminates good and poor outcome to first-line aromatase inhibitors in MBC patients [35]. 

Our results indicate a high heterogeneity in gene expression in CTCs, and this is in accordance with many previous studies highlighting the high heterogeneity of these cells using different isolation and detection methods [1,2,14,16,18,22,28,36,37,38]. Our approach was based on testing multiple RNA-markers on CTC, so that we could increase the sensitivity of CTC detection. To achieve this, we developed and used multiplex RT-qPCR assays; multiplex molecular assays have the advantage that require limited amount of sample for many different analytes, while the cost and time of analysis is also reduced [35]. Multiplex RT-qPCR assays apply perfectly to CTC analysis, since the amount of available sample for analysis is usually very low and CTC are highly heterogeneous [16,34,39].

Univariate and multivariate analysis confirmed the prognostic value of the expression of at least one of the following four gene expression profiles: *CK-19*, *CD44^high^/CD24^low^*, *ALDH1^high^/CD24^low^* and overexpression of *HER2* in the EpCAM^(+)^ CTC fraction for OS. Recently, by using an RT-PCR assay analyzing a 46-gene panel it was reported that only 14 genes were identified as significantly differentially expressed between CTC-positive and CTC-negative patients, and only four of these genes (*CK-19*, *EPCAM*, *CDH1* and *SCGB2A2*) were significantly differential expressed between the responders and non-responders [40]. Gene expression in CTC could further support the discovery of therapeutic predictors and is very promising for real-time identification of emerging resistance mechanisms in MBC patients [39]. Based on the fact that CTCs represent a rare population, combination of gene expression markers provides CTC detection and molecular characterization, even in cases where only one CTC is detected [41].

Survival and univariate analysis revealed that patients whose EpCAM^(+)^ CTCs were *CK-19^(+)^* or *HER2^(+)^*, had significantly shorter OS. The presence of *CK-19^(+)^* CTCs was of prognostic significance in early breast cancer patients [14]. The presence of *CK-19^(+)^* CTCs after the completion of chemotherapy is associated with increased risk of late relapse [36] and poor survival [37] in MBC. In early breast cancer the detection of *CK-19^(+)^* CTCs and *HER2^(+)^* CTCs is associated with shorter disease-free survival [14]. CTCs in women with HER2-negative breast cancer could acquire a HER2+ subpopulation, that is more proliferative but not addicted to HER2, consistent with activation of multiple signaling pathways [38]. Georgoulias et al. [25], have shown that “secondary adjuvant” trastuzumab therapy based on the HER2 phenotype of CTCs could eliminate *CK-19*mRNA/*HER2*-positive CTCs [25], and that these patients had a prolonged disease free survival. We report that HR (*ESR1*, *PR*, *HER2*) are detected in EpCAM^(+)^ CTCs at different positivity rates. More specifically, *HER2* positivity rate was higher (17.4%) than *ESR1* (13.0%), and *PR* (8.70%). Our results are in concordance with those reported by the DETECT Study Group who have shown, that HER2 is more highly expressed in CTCs in relation to ESR1 and PR, even though a different CTC isolation method and different volume of PB was used [23]. All six patients that were positive for *HER2* expression in EpCAM^(+)^ CTCs were negative for HER2 amplification in the primary tumor. Previous studies have also shown that in advanced breast cancer a subset of patients with HER2-negative primary tumors develop HER2-positive CTCs during disease progression [42,43], even after several months of either endocrine treatment or chemotherapy [44], which is an important part of endocrine resistance [27]. 

Several studies have shown that the majority of CTCs are *ESR1*/*PR*-negative [23] regardless of ER and PR expression on the primary tumors. Our results indicate a high discrepancy (>60%) in the ESR1/PR status between the primary tumor and EpCAM^(+)^ CTCs, and that *ER*/*PR* are expressed at a very low percentage in CTCs in respect to paired primary tumors. In MBC patients with ER-positive primary tumors, it has been proposed that lack of *ESR1* expression in CTCs could be a possible mechanism of resistance to endocrine therapy [45], and that is also associated to increased migration and invasion [46]. In MBC patients with ER-positive disease, the presence of *ESR1* mutations in CTCs is one of the diverse mechanisms of acquired endocrine drug resistance and could explain failure to suppress ER signaling within CTCs after 3 weeks of endocrine therapy [47]. Another study has shown that in MBC *ESR1* mutations were absent in primary tumor tissue samples and were detected only in metastases obtained after CTC characterization [48]. Our group has previously shown that epigenetic silencing of *ESR1* through methylation was associated with lack of response to endocrine treatment [18].

CTCs positive for stem cell markers are chemo-resistant, and their presence independently predicts for unfavorable outcome in MBC [49]. In the present study, Kaplan-Meier and univariate analysis revealed that patients with a positive *CD44^high^/CD24^low^* or *ALDH1^high^/CD24^low^* profile in CTCs had significantly shorter OS. Our findings are in agreement with previous studies, showing that the presence of HER2-positive CTCs co-expressing a breast cancer stem cell profile (HER2+/CD44^+^/CD24^(low)^) and elevated ALDH1 activity is related to aggressiveness and radioresistance [50]. 

## 5. Conclusions

Our results indicate that combined gene expression analysis in EpCAM^(+)^ CTCs provides prognostic information in MBC. These results need to be further confirmed in a prospective study, including a larger and well-defined patient cohort. 

## Figures and Tables

**Table 1:** Clinical characteristics of MBC patients.

Qualitative Variable	No. of Patients	Qualitative Variable	No. of Patients
Age		Tumor Grade	
<54	21 (45.7%)	II	25 (54.3%)
≥54	23 (50.0%)	III	15 (32.6%)
Unknown	2 (4.3%)	Unknown	6 (13.0%)
*Size*		*LN*	
<2 cm	9 (19.6%)	N0	13 (28.3%)
2–5 cm	27 (5.7%)	N1	12 (26.1%)
≥5 cm	8 (17.4%)	N2	7 (15.2%)
Unknown	2 (4.30%)	N3	12 (26.1%)
		Unknown	2 (4.30%)
ER		PR	
Positive	34 (73.9%)	Positive	30 (65.2%)
Negative	9 (19.6%)	Negative	13 (28.3%)
Unknown	3 (6.50%)	Unknown	3 (6.50%)
HER2		Death	
Positive	9 (19.6%)	Death	32 (69.6%)
Negative	34(73.9%)	Alive	14 (30.4%)
Unknown	3 (6.50%)		
**Metastasis**			
Bone-Lung	5 (10.9%)	Liver	3 (6.50%)
Lung	6 (13.0%)	Liver-bone	4 (8.70%)
Bone	24 (52.2%)	Liver-lung-bone	2 (4.30%)
Brain	1 (2.20%)	Unknown	1 (2.20%)
Therapy			
Chemo + hormono	9 (19.6%)		
chemo	37(80.4%)		

**Table 2:** Cox proportional analysis for the prediction of OS. Bold values indicates statistical significance at the *p* < 0.05 level.

Overall Survival (OS)						
	*Univariate Analysis*			*Multivariate Analysis*		
Covariant	HR ^a^	95% CI ^b^	*p*-Value ^c^	HR ^a^	95% CI ^b^	*p*-Value ^c^
Gene expression in EpCAM^(+)^ CTCs ^d^ (Yes vs. No)	3.403	1.645–7.040	**0.001**	4.930	1.828–13.300	**0.002**
Age (<54 vs. ≥ 54)	0.939	0.452–1.951	0.867	0.526	0.212–1.307	0.166
Nodes (N0 vs. N1 vs. N2 vs. N3)	1.161	0.856–1.576	0.337	1.205	0.786–1.846	0.392
Tumour Size (T1 vs. T2 vs. T3)	0.873	0.514–1.484	0.873	0.586	0.290–1.184	0.136
Grade (II vs. III)	2.253	1.080–4.702	**0.030**	3.547	1.430–8.799	**0.006**
ER (Yes vs. No)	0.294	0.127–0.681	**0.004**	0.320	0.086–1.196	0.090
PR (Yes vs. No)	0.697	0.318–1.528	0.367	0.783	0.218–2.815	0.707
HER2 (Yes vs. No)	0.613	0.230–1.632	0.327	1.323	0.342–5.114	0.684

^a^ Hazard ratio, ^b^ Confidence Interval of the estimated HR, ^c^ Test for trend, ^d^ *CK-19, CD44^high^/CD24^low^, ALDH1^high^/CD24^low^, HER2.*

## Data Availability

The data presented in this study are available on request from the corresponding author. The data are not publicly available due to ethical restrictions.

## Supplementary Materials

The following are available online at https://www.mdpi.com/2075-4418/11/3/513/s1, Supl. File 1: Analytical validation of the multiplex RT-qPCR assay for *ESR1*, *PR*, *HER2*, *EGFR*, Figure S1: Kaplan–Meier estimates for OS for: (**a**) *CK-19*; (**b**) *CD44^high^/CD24^low^*; (**c**) *ALDH1^high^/CD24^low^*; (**d**) *HER2* overexpression in the EpCAM^(+)^ CTC fraction of MBC patients with bone metastasis. Figure S2: Kaplan–Meier estimates for OS for: (**a**) *CK-19*; (**b**) *CD44^high^/CD24^low^*; (**c**) *ALDH1^high^/CD24^low^*; (**d**) *HER2* overexpression in the EpCAM^(+)^ CTC fraction of MBC patients that received only chemotherapy. Table S1: Gene expression in EpCAM^(+)^ cells in MBC patients (*n* = 46) in respect to survival status. Table S2: Comparison of *ESR1*, *PR*, *HER2* expression in EpCAM^(+)^ CTCs and corresponding primary tumors. Table S3: Gene expression analysis in CTCs and localization of metastasis.

## Abbreviations

*ALDH1*	aldehyde dehydrogenase 1 family member A1
*CD24*	antigen (small cell lung carcinoma cluster 4 antigen)
*CD44*	antigen (homing function and Indian blood group system)
*CK-19*	cytokeratin 19
CTCs	circulating tumor cells
DTCs	disseminated tumor cells
EGFR	epidermal growth factor receptor
EMT	epithelial to mesenchymal-like
ESR1	estrogen receptor 1
HD	healthy donors
HR	Hormonal receptors
*HER2*	erb-b2 receptor tyrosine kinase 2
*HPRT*	hypoxanthine phosphoribosyltransferase 1
MBC	metastatic breast cancer
mCRPC	metastatic castration resistant prostate cancer
MET	mesenchymal to epithelial-like
OS	overall survival
PR	progesterone receptor
*TWIST1*	twist family bHLH transcription factor 1
