# Supplementary material for: RNA-Based CTC Analysis Provides Prognostic Information in Metastatic Breast Cancer
Source: Diagnostics (Basel). 2021 Mar 14;11(3):513. doi: 10.3390/diagnostics11030513 (PMC7998407; doi:10.3390/diagnostics11030513)
Supplement: Supplementary file 1 [file diagnostics-11-00513-s001.zip › Figure S1.pptx]

## Slide 1
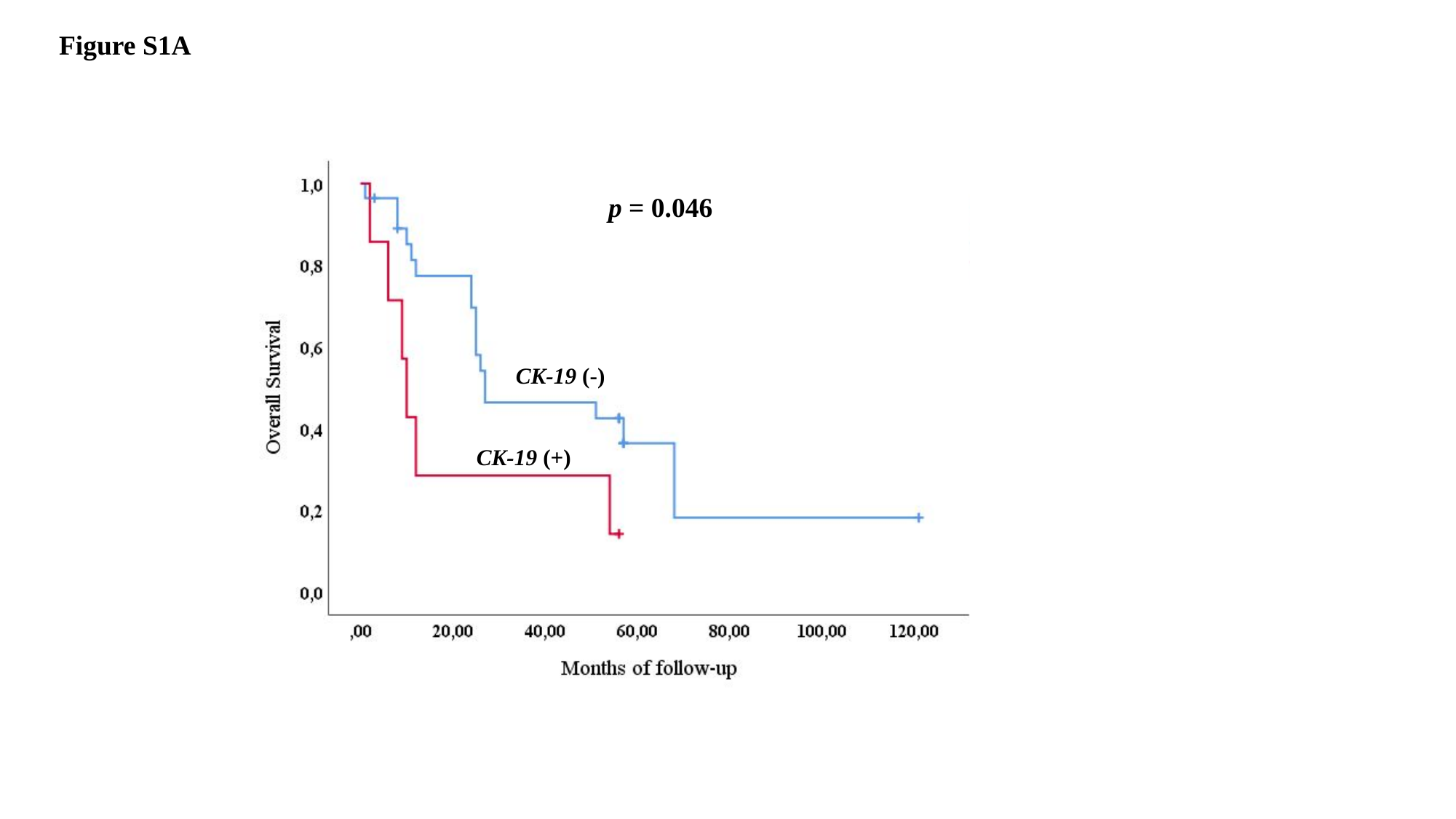

Figure S1A
p = 0.046
CK-19 (-)
CK-19 (+)

## Slide 2
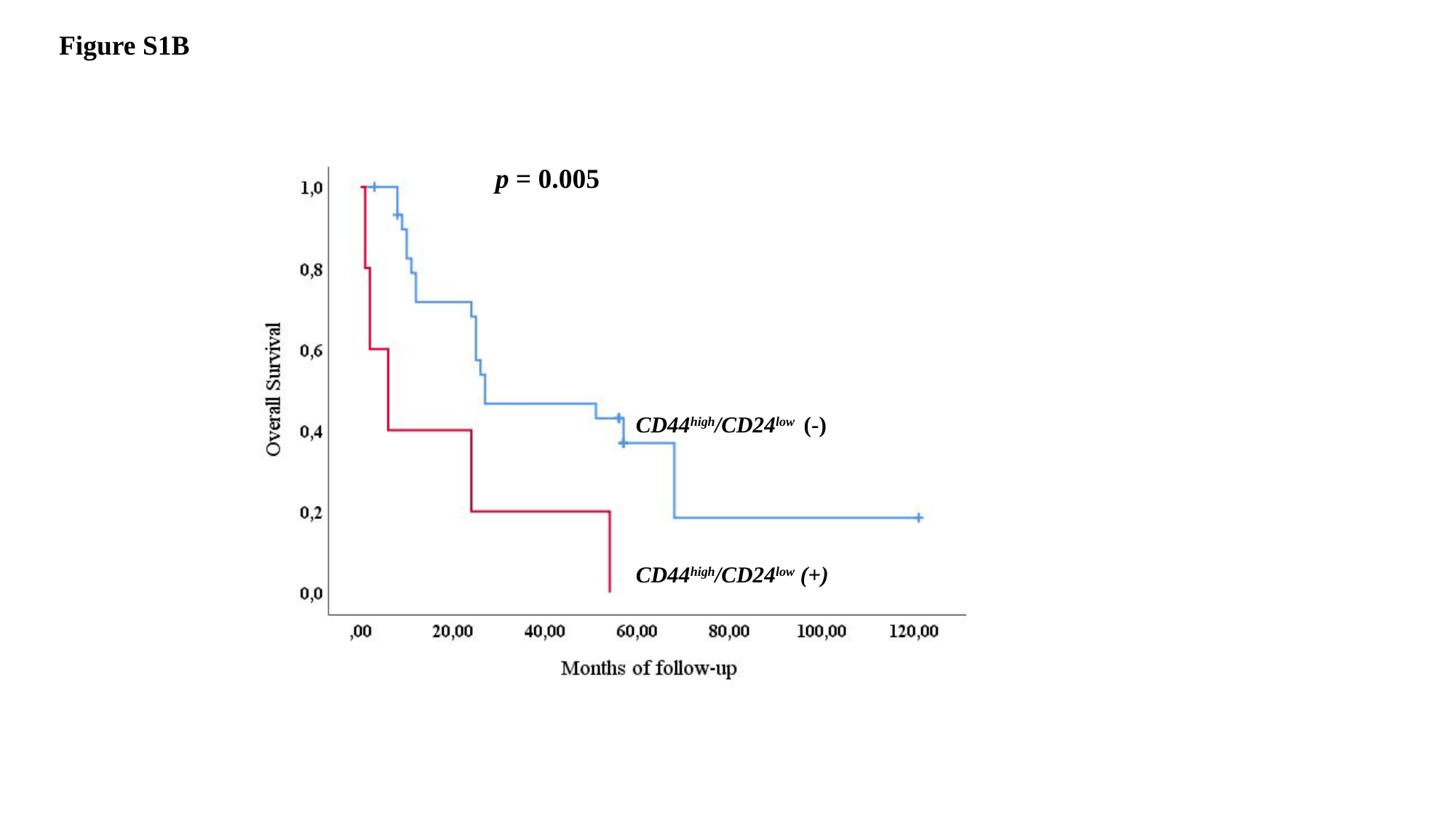

Figure S1B
p = 0.005
CD44high/CD24low (-)
CD44high/CD24low (+)

## Slide 3
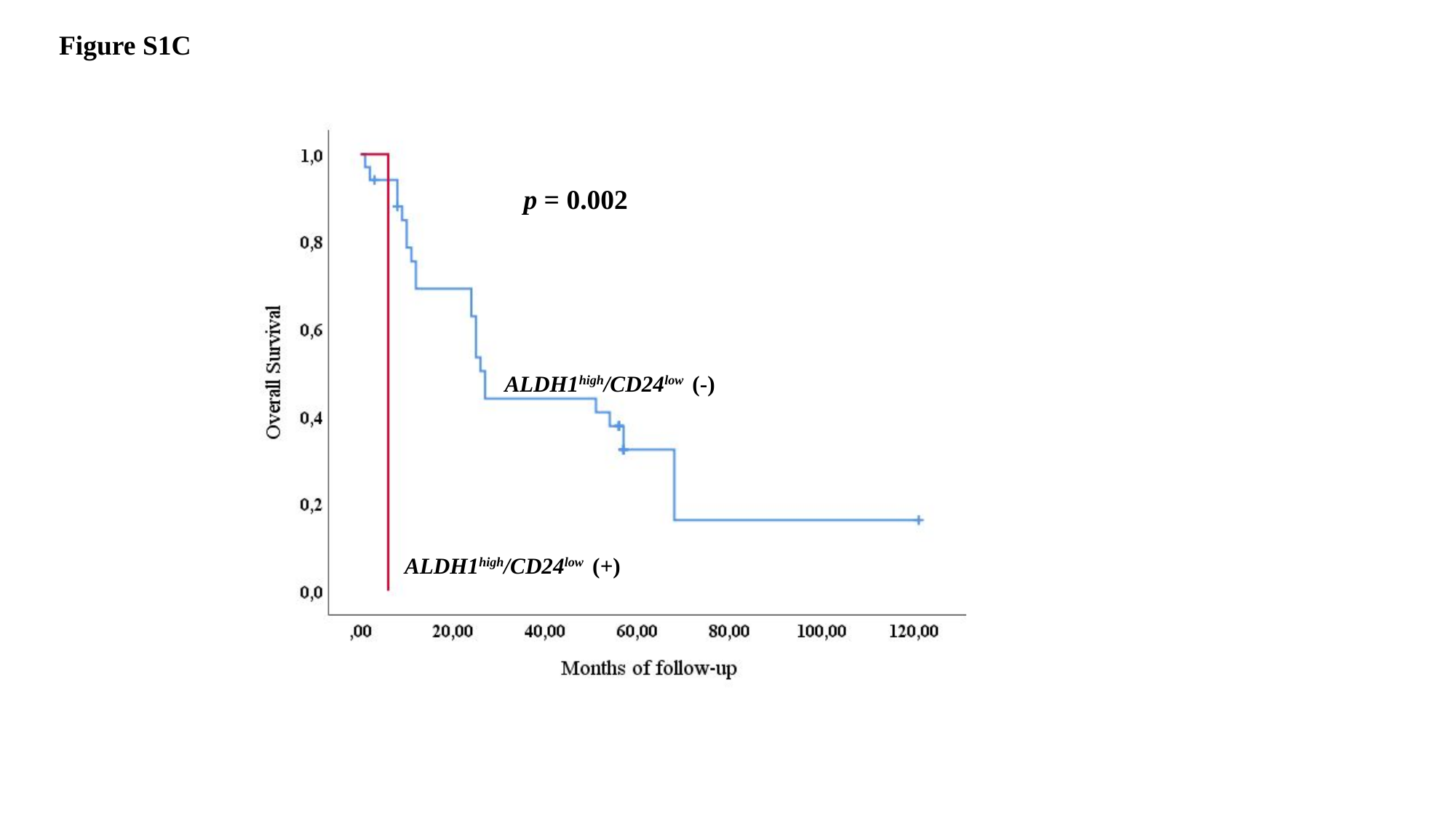

Figure S1C
p = 0.002
ALDH1high/CD24low (-)
ALDH1high/CD24low (+)

## Slide 4
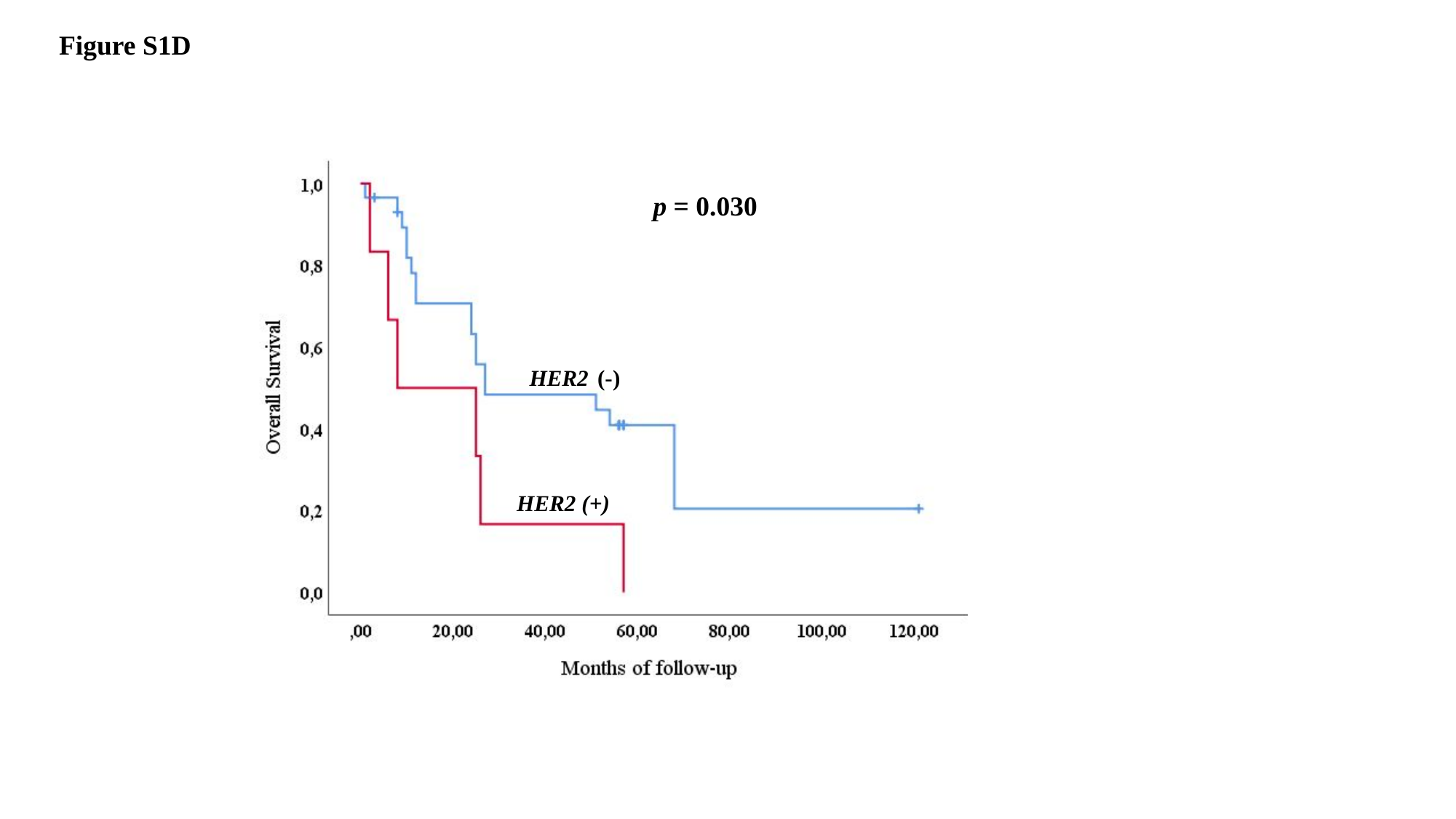

Figure S1D
p = 0.030
HER2 (-)
HER2 (+)
