# Supplementary material for: RNA-Based CTC Analysis Provides Prognostic Information in Metastatic Breast Cancer
Source: Diagnostics (Basel). 2021 Mar 14;11(3):513. doi: 10.3390/diagnostics11030513 (PMC7998407; doi:10.3390/diagnostics11030513)
Supplement: Supplementary file 1 [file diagnostics-11-00513-s001.zip › Figure S2.pptx]

## Slide 1
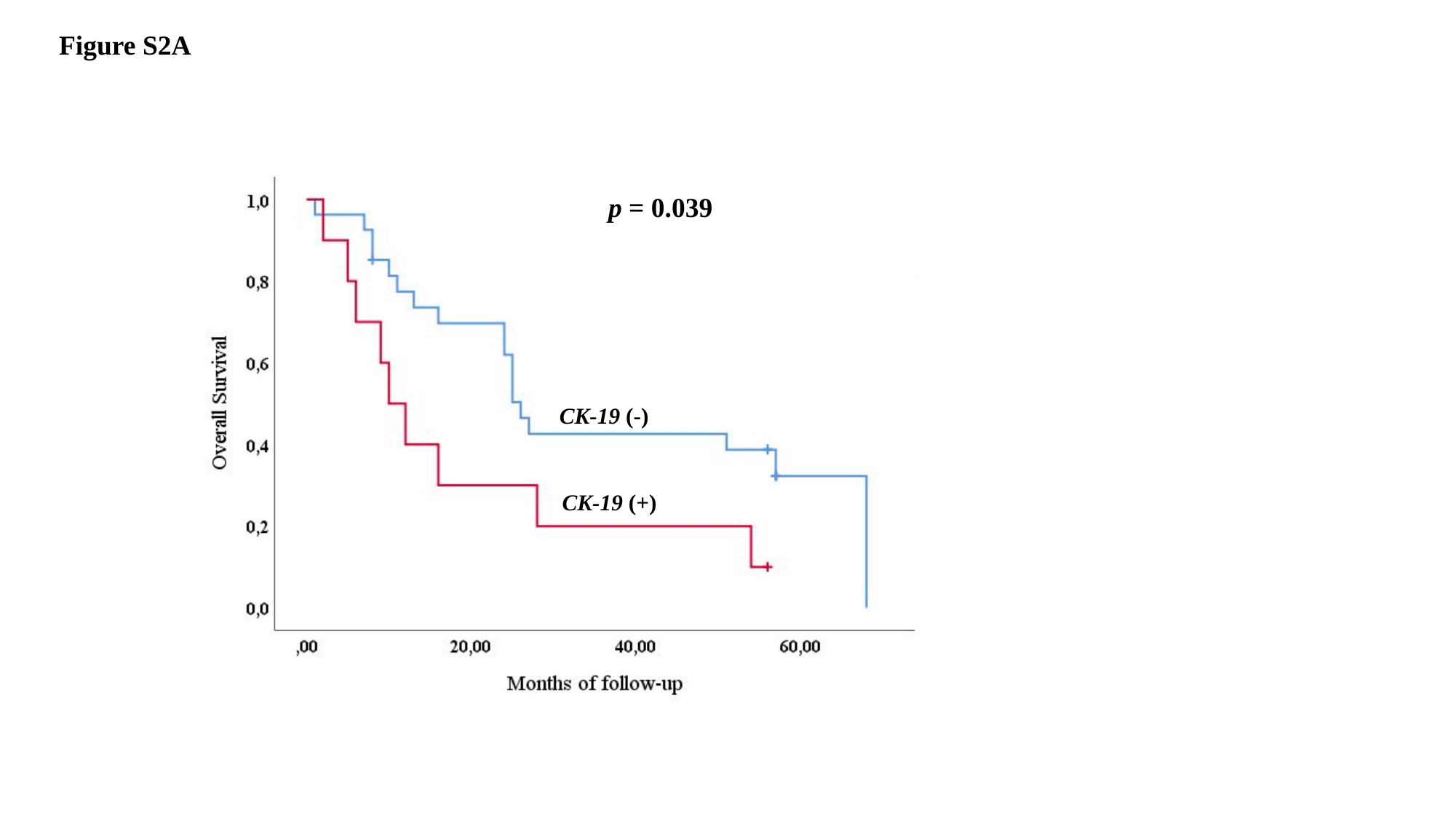

Figure S2A
p = 0.039
CK-19 (-)
CK-19 (+)

## Slide 2
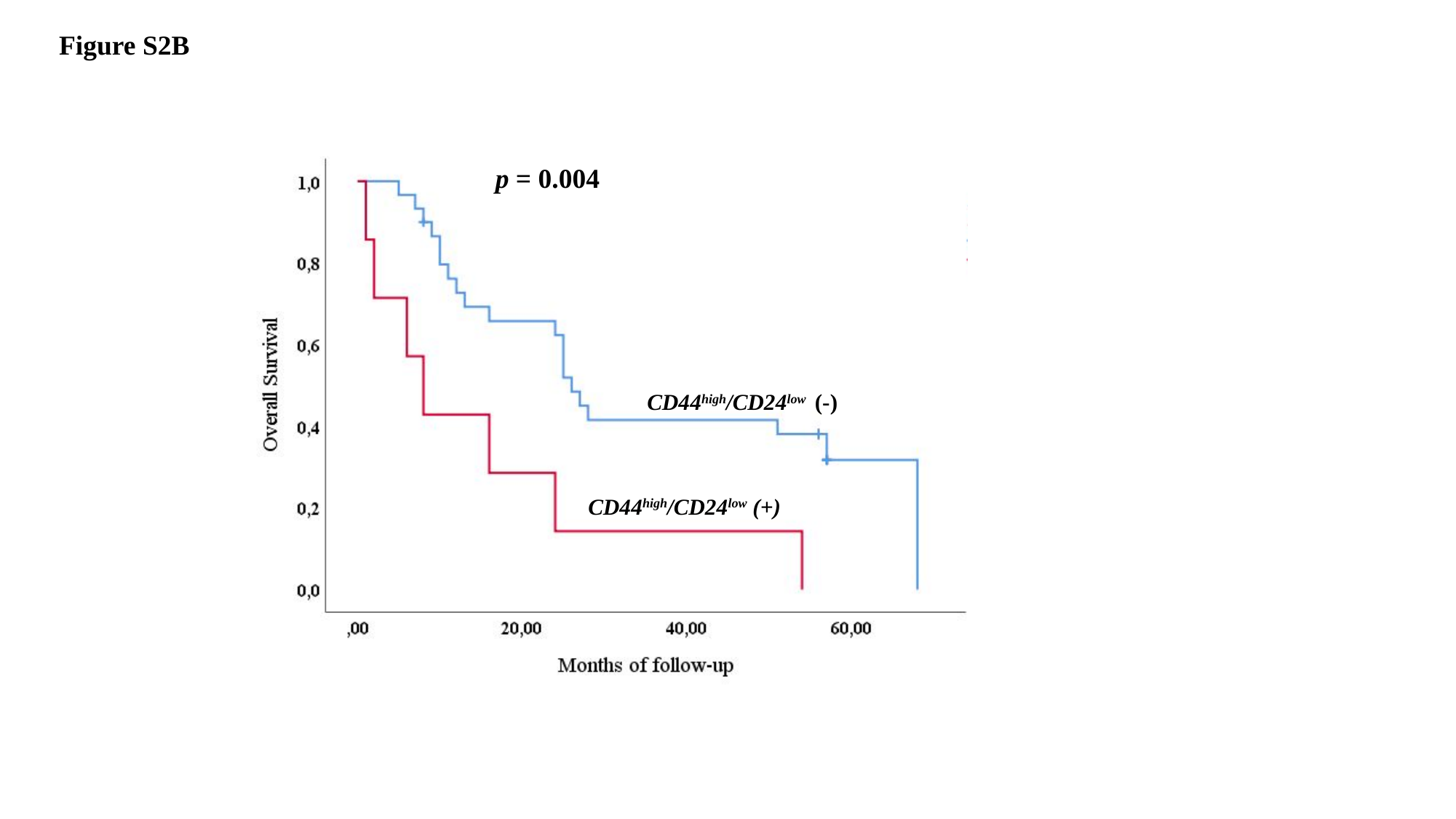

Figure S2B
p = 0.004
CD44high/CD24low (-)
CD44high/CD24low (+)

## Slide 3
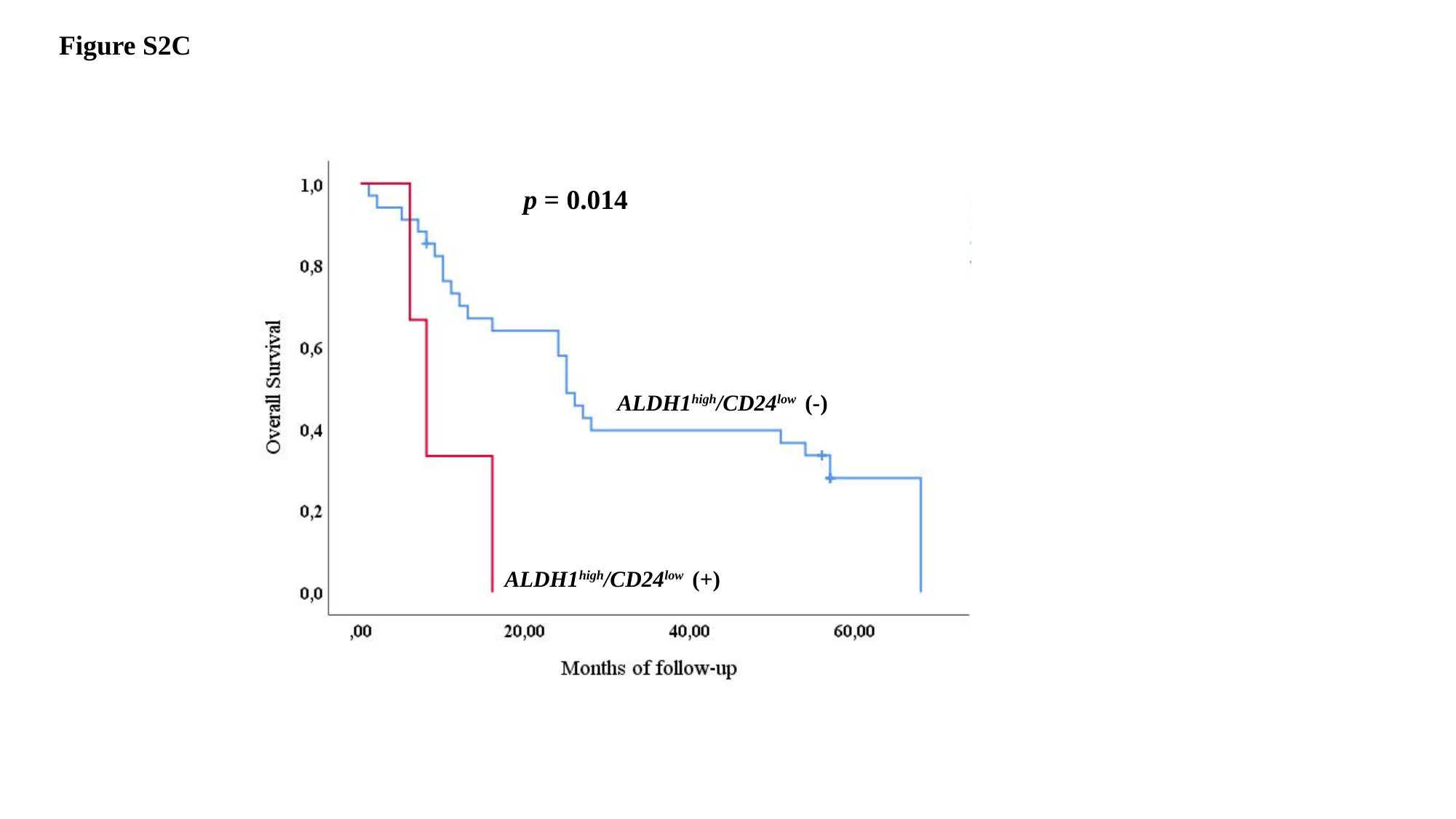

Figure S2C
p = 0.014
ALDH1high/CD24low (-)
ALDH1high/CD24low (+)

## Slide 4
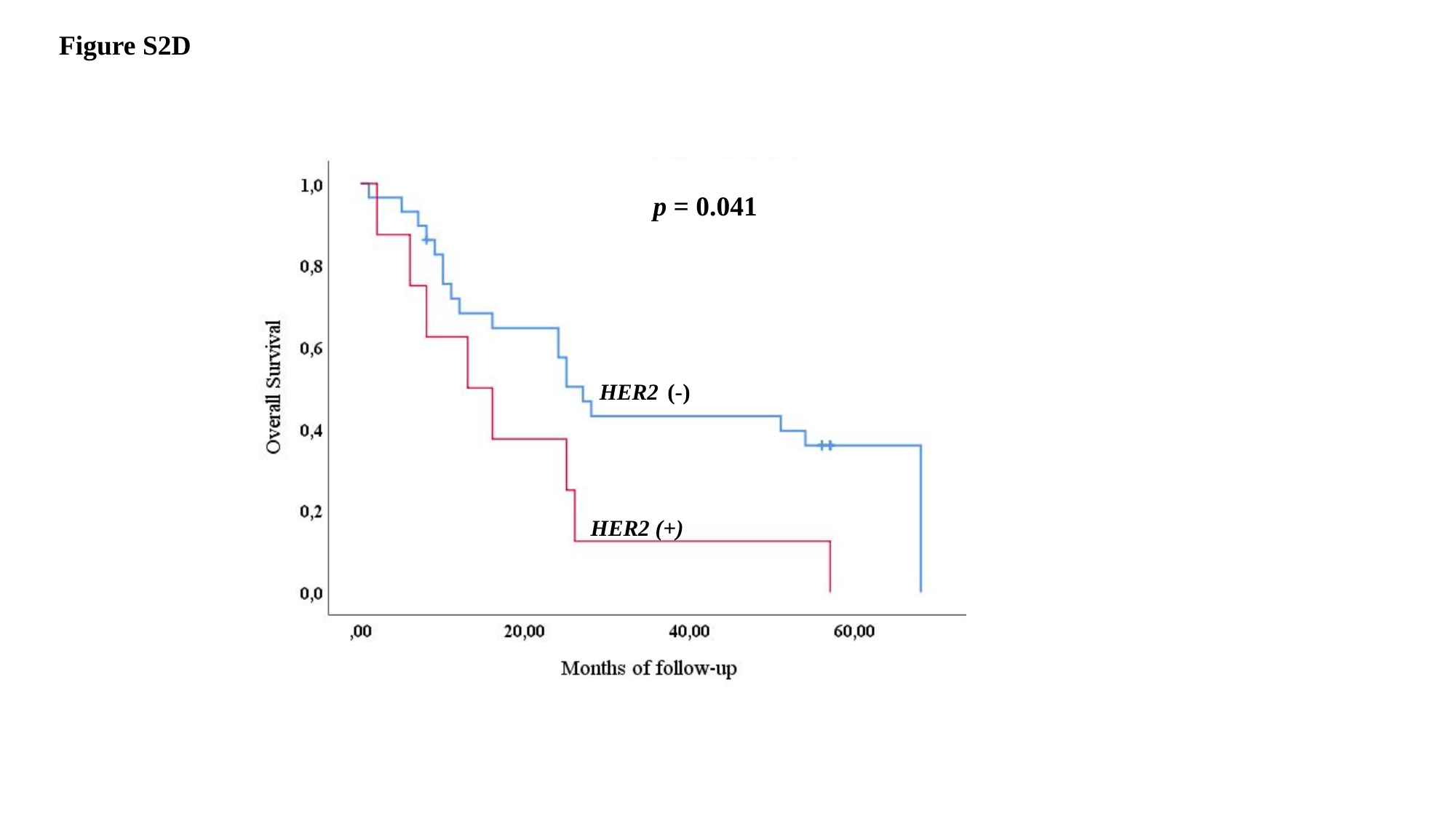

Figure S2D
p = 0.041
HER2 (-)
HER2 (+)
