# Supplementary material for: RNA-Based CTC Analysis Provides Prognostic Information in Metastatic Breast Cancer
Source: Diagnostics (Basel). 2021 Mar 14;11(3):513. doi: 10.3390/diagnostics11030513 (PMC7998407; doi:10.3390/diagnostics11030513)
Supplement: Supplementary file 1 [file diagnostics-11-00513-s001.zip › Supl. Tables.docx]

**Table S1.** Gene expression in EpCAM^(+)^ cells in MBC patients (*n* = 46) in respect to survival status. Bold values indicates statistical significance at the *p* < 0.05 level.

| **Gene target** | **RT-qPCR** | **Survival status** | | | ***p*^a^** |
| --- | --- | --- | --- | --- | --- |
|  |  | **Dead**  32(69.6%) | **Alive**  14(30.4%) | **Total**  **46** |  |
| ***CK-19*** | **Positive** | 9 | 1 | 10 | 0.143 |
|  | **Negative** | 23 | 13 | 36 |  |
| ***ER*** | **Positive** | 5 | 1 | 6 | 0.651 |
|  | **Negative** | 27 | 13 | 40 |  |
| ***PR*** | **Positive** | 3 | 1 | 4 | N.S |
|  | **Negative** | 29 | 13 | 42 |  |
| ***HER2*** | **Positive** | 8 | 0 | 8 | 0.085 |
|  | **Negative** | 24 | 14 | 38 |  |
| ***CD44^high^/CD4^low^*** | **Positive** | 7 | 0 | 7 | 0.083 |
|  | **Negative** | 25 | 14 | 39 |  |
| ***ALDH1^high^/CD24^low^*** | **Positive** | 3 | 0 | 3 | 0.543 |
|  | **Negative** | 29 | 14 | 43 |  |
| ***TWIST1*** | **Positive** | 1 | 0 | 1 | N.S |
|  | **Negative** | 31 | 14 | 45 |  |
| **Combination of all**  **gene targets** | **Positive** | 21 | 3 | **24** | **0.010** |
|  | **Negative** | 11 | 11 | **22** |  |

^a^Fischer-Exact test

**Table S2.** Comparison of *ESR1*, *PR*, *HER2* expression in EpCAM^(+)^ CTCs and corresponding primary tumors.

| **ER expression in primary tumor (IHC)** | ***ESR1* expression in EpCAM^(+)^CTCs**  **(RT-qPCR)** | | | |
| --- | --- | --- | --- | --- |
|  |  | Positive | Negative | Total |
|  | Positive | 4 | 30 | 34 |
|  | Negative | 1 | 8 | 9 |
|  | Total | 5 | 38 | 43 |
|  | **Concordance: 12/43 (27.9%),** (p^a^=1.000) | | | |
| **PR expression in primary tumor (IHC)** | ***PR* expression in EpCAM^(+)^CTCs**  **(RT-qPCR)** | | | |
|  |  | Positive | Negative | Total |
|  | Positive | 3 | 27 | 30 |
|  | Negative | 1 | 12 | 13 |
|  | Total | 4 | 39 | 43 |
|  | **Concordance: 15/43 (34.9%),** (p^a^=1.000) | | | |
| **HER2 amplification in primary tumor (IHC/FISH)** | ***HER2* expression in EpCAM^(+)^CTCs**  **(RT-qPCR)** | | | |
|  |  | Positive | Negative | Total |
|  | Positive | 0 | 9 | 9 |
|  | Negative | 6 | 28 | 34 |
|  | Total | 6 | 37 | 43 |
|  | **Concordance: 28/43 (65.1%),** (p^a^=0.315) | | | |

^a^Fischer-Exact test

**Table S3.** Gene expression analysis in CTCs and localization of metastasis.

| **Gene target** | **RT-qPCR** | **Metastatic site** | | | **P^a^** |
| --- | --- | --- | --- | --- | --- |
|  |  | **Bone**  35(76.1%) | **Other**  10(21.7%) | Total  45 |  |
| ***CK-19*** | **Positive** | 7 | 2 | 9 | 1.00 |
|  | **Negative** | 28 | 8 | 36 |  |
| ***ER*** | **Positive** | 5 | 1 | 6 | 1.00 |
|  | **Negative** | 30 | 9 | 39 |  |
| ***PR*** | **Positive** | 4 | 0 | 4 | 0.561 |
|  | **Negative** | 31 | 10 | 41 |  |
| ***HER2*** | **Positive** | 6 | 2 | 8 | 1.00 |
|  | **Negative** | 29 | 8 | 37 |  |
| ***CD44^high^/CD4^low^*** | **Positive** | 5 | 2 | 7 | 0.642 |
|  | **Negative** | 30 | 8 | 38 |  |
| ***ALDH1^high^/CD24^low^*** | **Positive** | 1 | 2 | 3 | 0.119 |
|  | **Negative** | 34 | 8 | 42 |  |
| ***TWIST1*** | **Positive** | 0 | 1 | 1 | 0.272 |
|  | **Negative** | 35 | 9 | 44 |  |
| **Combination of all**  **gene targets** | **Positive** | 17 | 6 | 23 | 0.722 |
|  | **Negative** | 18 | 4 | 22 |  |
