# Supplementary material for: RNA-Based CTC Analysis Provides Prognostic Information in Metastatic Breast Cancer
Source: Diagnostics (Basel). 2021 Mar 14;11(3):513. doi: 10.3390/diagnostics11030513 (PMC7998407; doi:10.3390/diagnostics11030513)
Supplement: Supplementary file 1 [file diagnostics-11-00513-s001.zip › SUPPLEMENTARY FILES.docx]

**SUPPL FILE 1**

**Analytical validation of the multiplex RT-qPCR assay for *ESR1*, *PR*, *HER2*, *EGFR***

The analytical specificity of the developed multiplex RT-qPCR assay for *ESR1*, *PGR*, *HER2*, and EGFR was evaluated individually for each assay. This was checked by using all oligonucleotides in a common master mix in four different reactions in the presence of one individual gene target. The assay is highly specific, since each primer pair and dual hybridization probe pair amplifies specifically only the corresponding target sequence and is detected only in the corresponding wavelength (**Supplementary Fig. S3A**). The analytical sensitivity of each individual assay in this multiplex RT-qPCR was determined for each individual gene target using a calibration curve. These calibration curves were generated using serial dilutions of individual gene-specific external standards in triplicate for each concentration, ranging from 10^5^copies/μL to 10 copies/μL. The developed multiplex RT-qPCR assay showed linearity over the entire quantification range and correlation coefficients greater than 0.99 in all cases, indicating a precise log-linear relationship. The limit of detection (LOD) was found to correspond to 3 copies/μL while the limit of quantitation (LOQ) was 9 copies/μL (**Supplementary Fig. S3B**). Repeatability or intra–assay variance of the multiplex RT-qPCR, was evaluated by repeatedly analyzing 2 external calibrators corresponding to 100 and 1000 copies/μL, in the same assay, in 3 parallel determinations (**Supplementary Table S4**). Reproducibility or inter–assay variance, was evaluated by analyzing the same cDNA sample, representing 100 SKBR-3 and T47D cells on 5 separate assays performed in 5 different days (**Supplementary Table S4**).


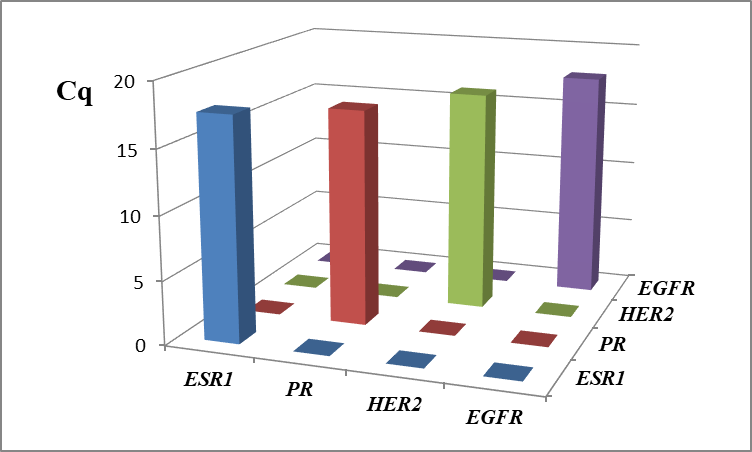


a)

b)

**
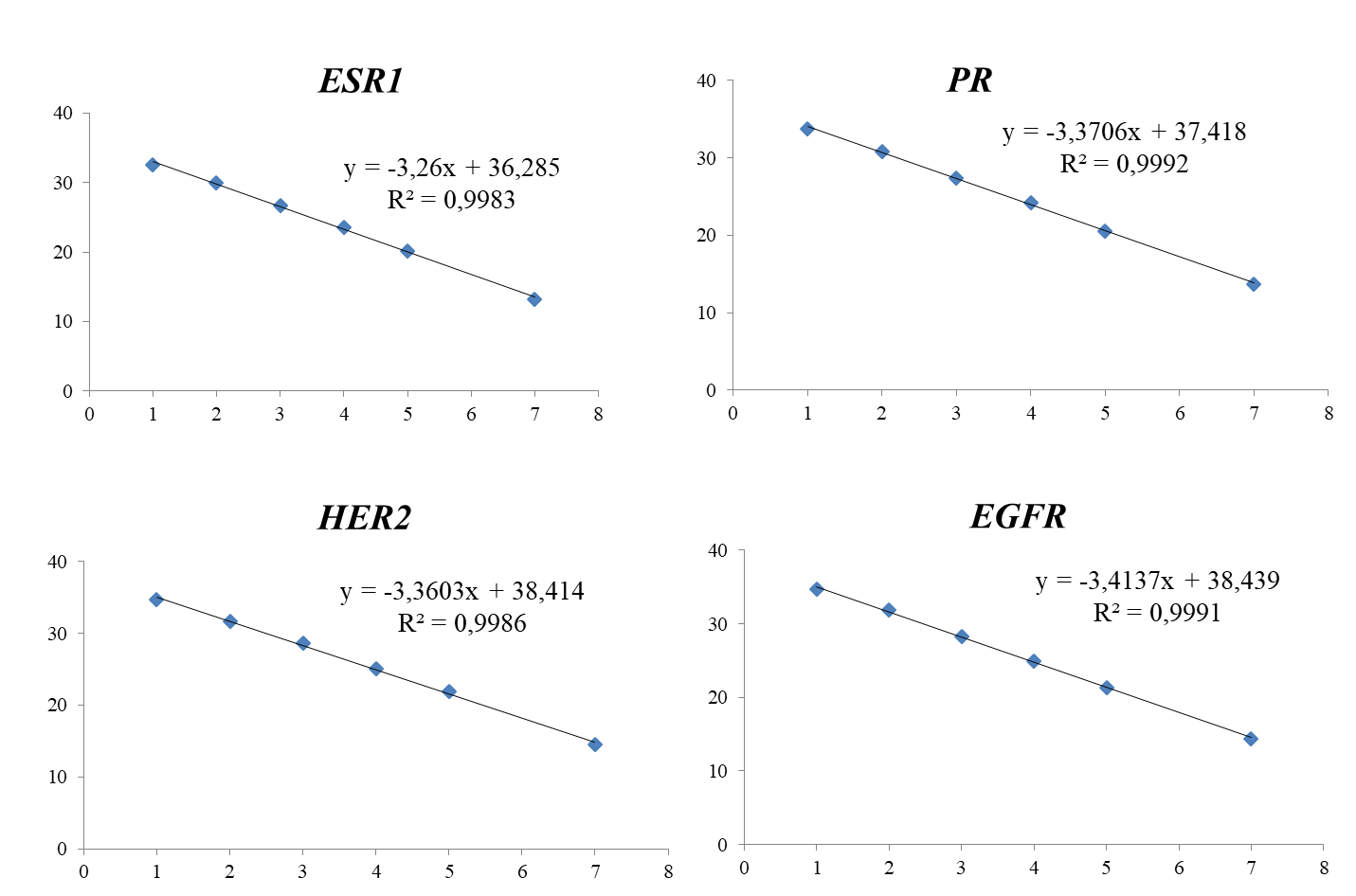
**

**Figure S3.** Analytical validation of the multiplex RT-qPCR for *ESR1*, *PR*, *HER2*, *EGFR* (all measured in triplicate) (a) analytical specificity, (b) RT-qPCR calibration curves (copies/μL)

**Table S4.** Multiplex RT-qPCR for *ESR1*, *PGR, HER2, EGFR*: intra- (n=3) and inter-assay precision (n=5).

| **Gene target** | **Multiplex RT-qPCR Assay Precision** | |
| --- | --- | --- |
| ***ESR1*** | ***Intra-assay precision (n=3)*** | |
| **Copies/μL** | **Cq (SD)** | **CV%** |
| 100 | 28.87 (0.18) | 0.62 |
| 1000 | 26.70 (0.083) | 0.31 |
|  | ***Inter- assay precision (n=5)*** | |
| 100 SKBR3+T47D | 18.54 (0.15) | 0.83 |
| ***PR*** | ***Intra-assay precision (n=3)*** | |
| **Copies/μL** | **Cq (SD)** | **CV%** |
| 100 | 28.77(0.21) | 0.73 |
| 1000 | 26.34 (0.058) | 0.22 |
|  | ***Inter- assay precision (n=5)*** | |
| **100 SKBR3+T47D** | 18.18 (0.24) | 1.3 |
| ***HER2*** | ***Intra-assay precision (n=3)*** | |
| **Copies/μL** | **Cq (SD)** | **CV%** |
| 100 | 30.02 (0.09) | 0.30 |
| 1000 | 28.33 (0.04) | 0.14 |
|  | ***Inter- assay precision (n=5)*** | |
| **100 SKBR3+T47D** | 19.75 (0.16) | 0.79 |
| ***EGFR*** | ***Intra-assay precision (n=3)*** | |
| **Copies/μL** | **Cq (SD)** | **CV%** |
| 100 | 30.74 (0.09) | 0.30 |
| 1000 | 28.20 (0.12) | 0.43 |
|  | ***Inter- assay precision (n=5)*** | |
| **100 SKBR3+T47D** | 24.83 (0.16) | 0.64 |
